# Fentanyl Versus Ondansetron for Shivering Prevention in Cesarean Section: A Comparative Study

**DOI:** 10.7759/cureus.46817

**Published:** 2023-10-10

**Authors:** Shaimaa H Hasan, Reabar Haji Qadir, Haider N Mohammed

**Affiliations:** 1 Anesthesia Department, College of Health Sciences, University of Duhok, Duhok, IRQ

**Keywords:** anesthesia, cesarean section, spinal anesthesia, ondansetron, intrathecal fentanyl

## Abstract

Background and purpose: The incidence of postoperative shivering (PS) following intrathecal anesthesia is a common complication, with potential negative impacts on patient outcomes. This study aims to evaluate the effectiveness of intrathecal fentanyl versus intravenous ondansetron in preventing post-spinal anesthesia shivering in cesarean section patients.

Experimental approach: A randomized controlled trial was conducted from July 2021 to April 2023, involving pregnant women scheduled for cesarean section under intrathecal anesthesia. The participants were divided into three groups: group F received intrathecal fentanyl (15 μg) with spinal anesthesia, while group O received intravenous ondansetron (8 mg) added to the usual saline solution. The control group (group C) received only intravenous fluid before spinal anesthesia and intrathecal bupivacaine without fentanyl. Shivering occurrences were observed and recorded during the procedure.

Key results: The incidence of shivering was significantly lower in the fentanyl and ondansetron groups compared to the control group (p=0.0123). Both intrathecal fentanyl and intravenous ondansetron administration showed effectiveness in reducing shivering during spinal anesthesia.

Conclusion: The administration of intrathecal fentanyl and intravenous ondansetron significantly reduced the occurrence of shivering during spinal anesthesia in cesarean section patients. This study contributes to advancing knowledge in the field by providing evidence of the preventative effects of these medications on post-spinal anesthesia shivering.

## Introduction

Anesthesia complications such as postoperative shivering (PS) are common; under general anesthesia, they have been estimated to range from 20% to 70%. Shivering is thought to increase the risk of hypoxemia, increase oxygen use, and increase postoperative complications. Hypothermia typically causes shivering. In the perioperative period, it happens even to normothermic individuals. There is not enough knowledge on the cause of shivering [1]. An evaluation of the enumeration of literature published on the subject is necessary due to the significance of this complication as a postoperative complication and the paucity of data regarding its genesis and management. Shivering is a common and difficult adverse reaction to anesthesia and targeted temperature control [2].

Shivering is a phenomenon characterized by involuntary oscillatory contractions of skeletal muscles. After peripheral vasoconstriction, shivering is the body's physiological response to being exposed to cold temperatures [3]. Postoperative shivering (PS) is an uncontrollable, rhythmic muscle movement that occurs in the initial stages of anesthesia recovery. Facial, jaw, or head movement disorders, as well as muscle hyperactivity, are referred to as shivering. The post-anesthesia care unit frequently witnesses this phenomenon. According to earlier research, incidence after general anesthesia ranged from 5% to 65% and following epidural treatments from 30% to 33%. In a recent meta-analysis, the overall shivering incidence was 34%. The research found a distinct incidence of non-thermoregulatory shivering in normothermic postoperative patients in studies including surgical patients rather than volunteers. A non-temperature-dependent impact of isoflurane anesthesia was a tonic stiffening pattern of muscle activation. A spontaneous electromyography clonus was another pattern that was seen, and hypothermia and residue isoflurane end-tidal concentrations of 0.4%-0.2% were necessary. The following shivering score, which evaluates the severity of shivering, is provided by Mathews et al. [4]. No shivering is indicated by a score of 0, minor facial and neck fasciculation and electrocardiographic (ECG) abnormalities are indicated by a score of 1, a visible tremor is indicated by a score of 2, and severe muscular activity involving the entire body is indicated by a score of 3 [4]. Hypothermia typically causes PS and can also occur in normothermic individuals in the perioperative period. There is not enough knowledge regarding the cause of shivering [5].

In transurethral resection of the prostate, intrathecal fentanyl may lessen the frequency and intensity of shivering during spinal anesthesia. All the same, this study aims to evaluate the effectiveness of intrathecal fentanyl versus intravenous ondansetron in preventing post-spinal anesthesia shivering in cesarean section patients.

## Materials and methods

Location of the study

Duhok Hospital is the study's location. Each participant in the study gave their written informed consent. This has ethical clearance. The research was performed in accordance with the principle of the College of Health Sciences and was approved by the Ethics Committee of Duhok University and the Duhok Directorate General Health on April 2, 2023 (approval number: 15092021-9-1 R1). Accordingly, the researcher was given the right to publish the results of the study.

Design and sample size of the current study

Inclusion Criteria

Patients who were 18 to 41 years old, with American Society of Anesthesiologists (ASA) physical status I and II, and who underwent cesarean section under spinal anesthesia were included.

Exclusion Criteria

Patients who refused to participate; who are not candidates for spinal anesthesia (e.g., those with infection, coagulopathy, or vertebral column deformity); who are hypersensitive to the medications being used (ondansetron is used as a preoperative medication); suffering from severe cardiac, pulmonary, renal, hepatic, and neuromuscular diseases and diabetes mellitus; taking selective serotonin reuptake inhibitor antidepressants or migraine medication; suffering from morbid obesity and pregnancy-induced hypertension; and pregnant woman with a high-risk pregnancy were excluded [6].

Current Prospective Study

A clinical trial was performed on 250 full-term pregnant women from July 2021 to April 2023. Cases were divided randomly into three groups, and the groups received 500 mL of normal saline at room temperature for 30 minutes preoperatively.

Study Design

The study is a randomized, prospective, double-blind clinical investigation. In group O, ondansetron (8 mg) had been added to their normal saline, while group F received 15 μg of fentanyl intrathecally with spinal anesthesia. Both groups received 0.5% heavy bupivacaine intrathecally for spinal anesthesia (15 μg of fentanyl intrathecally and 8 mg of intravenous ondansetron). The control group (group C) received only intravenous fluid before spinal anesthesia and intrathecal bupivacaine without fentanyl. The severity and frequency of shivering had been evaluated and recorded intraoperatively.

Data analysis and processing

Before being input into the computer, each filled-out survey was double-checked for accuracy, coded, and forwarded to Statistical Package for the Social Sciences (SPSS) version 19.0 (IBM SPSS Statistics, Armonk, NY) for analysis. To define the characteristics of the demographics, descriptive statistics were used. The Shapiro-Wilk test was performed to check for data distributions, and Levene's non-parametric test, the Kruskal-Wallis test, and the chi-square test were used to determine the significant difference between percentages. A two-tailed p-value of <0.05 was regarded as statistically significant.

## Results

Demographic result

Of the patients, 27.2% were between 18 and 25 years, 58.8% were between 26 and 36 years, and 14% were between 36 and 41 years. Most of the patients were overweight (98.48%), and 1.51% had normal weight (Table 1).

**Table 1:** Demographic characteristics of the patients in the current study BMI: body mass index

Parameter (N=250)		Number (%)
Age (years)	18-25	68 (27.2%)
	26-34	147 (58.8%)
	35-41	35 (14 %)
BMI (kg/m^2^)	Underweight (<19 kg/m^2^)	0 (0%)
	Normal (19-23 kg/m^2^)	4 (1.51%)
	Overweight (>24 kg/m^2^)	246 (98.48%)

Table 2 shows that the number of occurrences of shivering in the control group is significantly higher than in the fentanyl and ondansetron groups (p=0.0123).

**Table 2:** Comparison of study groups in terms of shivering occurrence

N=250	Group O	Group F	Group C	p-value
Yes	14 (19.5%)	20 (27.7%)	25 (37%)	0.0123
No	58 (80.5%)	52 (72.3%)	41 (62.12%)	0.001

Table 3 indicates that the use of 9 mg of bupivacaine showed a non-significant difference (p=0.987) in shivering between groups, while the other doses showed a significant difference, with 10, 11, 12, and 13 mg doses showing significantly higher shivering occurrence.

**Table 3:** Different doses of bupivacaine and the occurrence of shivering between groups

Bupivacaine dose		Group O (n=72)	Group F (n=72)	Group C (n=66)	p-value
9 mg	Yes	0 (0%)	0 (0%)	0 (0%)	0.987
	No	72 (100%)	72 (100%)	66 (100%)	
10 mg	Yes	20 (28.57%)	10 (14.28%)	40 (28.57%)	0.001
	No	52 (71.42%)	62 (85.71%)	26 (71.42%)	
11 mg	Yes	18 (27.27%)	8 (18.18%)	30 (45.45%)	0.001
	No	54 (72.72%)	64 (81.81%)	36 (54.54%)	
12 mg	Yes	24 (20%)	12 (10%)	36 (60%)	0.001
	No	48 (80%)	60 (90%)	30 (40%)	
13 mg	Yes	26 (33.33%)	0 (0%)	44 (66.66%)	0.001
	No	52 (66.66%)	72 (100%)	22 (33.33%)	

Table 4 indicates that the use of 10 mg of ephedrine showed a non-significant difference (p=0.056) in shivering between groups, while the other doses showed a significant difference, with 15, 20, 25, and 30 mg doses showing significantly higher shivering occurrence.

**Table 4:** Different doses of ephedrine and the occurrence of shivering between groups

Ephedrine dose		Group O (n=72)	Group F (n=72)	Group C (n=66)	p-value
10 mg	Yes	28 (28.57%)	28 (28.57%)	25 (35.71%)	0.056
	No	44 (71.43%)	44 (71.43%)	41 (64.29%)	
15 mg	Yes	16 (16.67%)	16 (16.67%)	22 (33.33%)	0.001
	No	56 (83.33%)	56 (83.33%)	44 (66.67%)	
20 mg	Yes	10 (10.34%)	27 (27.58%)	12 (18.18%)	0.0023
	No	62 (89.66%)	45 (72.42%)	54 (81.82%)	
25 mg	Yes	0 (0%)	12 (10%)	30 (57.14%)	0.001
	No	72 (100%)	60 (90%)	36 (42.86%)	
30 mg	Yes	20 (20%)	0 (0%)	30 (50%)	0.034
	No	52 (80%)	72 (100%)	36 (50%)	

## Discussion

Shivering is a common occurrence in the perioperative period, and it is often associated with hypothermia. While discomfort, disinhibited spinal reflexes, reduced sympathetic activity, and respiratory alkalosis have all been suggested as possible explanations of the phenomena, cold-induced thermoregulatory shivering is still the clearest etiology. According to conventional wisdom, post-anesthetic tremor results from the sudden dissipation of an aesthetic-induced thermoregulatory inhibition, which raises the shivering threshold toward normal [7]. Remifentanil is first removed more quickly than other opioids. Because the shivering threshold falls lower than body temperature during surgery, opioids prevent thermoregulatory responses, which prevents shivering. Because of the drug's kinetics, the threshold might instantly recover to normal following discontinuation. Shivering is brought on when the threshold rises during the recovery from general anesthesia more quickly than the rise in body temperature.

The second mechanism has to do with pain; in fact, remifentanil causes PS more frequently at high dosages than at low levels. This outcome is consistent with hyperalgesia brought on by high remifentanil dosages in earlier investigations. Patients who receive high doses of remifentanil are consequently susceptible to shivering following abrupt cessation. Shivering is an opiate withdrawal sign caused by acute tolerance, which is another mechanism. Remifentanil is a type of opioid with a short duration of action that has the potential to lead to tolerance to opioids and hyperalgesia, particularly when higher doses are given on a regular schedule. Remifentanil hyperalgesia may be avoided by ketamine, a common N-methyl-D-aspartate (NMDA) antagonist. At small doses, it is believed that the NMDA receptors play an important part in the development of tolerance to opioids. Remifentanil was thought to stimulate the NMDA receptors, as well as glycine, and pretreatment ketamine has been shown to be beneficial in the prevention of PS. Magnesium is likewise an NMDA receptor antagonist that is non-competitive, and intraoperative magnesium sulfate infusion decreases PS. Shivering after neuraxial anesthesia may have a distinct mechanism. Shivering occurs often during neuraxial (epidural and spinal anesthesia) and general anesthesia, with incidences of 40%-60% for regional anesthetic patients and up to 60% for general anesthetic patients. Compared to neuraxial anesthesia, general anesthesia causes more shivering. Spinal anesthesia impacts both central and peripheral thermoregulation by extending the inter-threshold range by increasing the sweating threshold and lowering the vasoconstriction and shivering thresholds, which could be impaired by general anesthesia [7].

Intraoperative hypothermia is a typical anesthesia practice concern. Like general anesthesia, regional anesthetic has an impact on the temperature-regulating mechanism. Ondansetron was used in a study to prevent post-anesthetic shivering in individuals receiving general anesthesia [5]. An effective 5-HT3 antagonist, ondansetron is typically prescribed to prevent and cure nausea and vomiting before, during, and after surgery. It is unclear how 5-HT3 antagonists regulate body temperature; however, it may be related to the preoptic anterior hypothalamic region's suppression of serotonin uptake [5]. In a study by Powell and Buggy [8], it was demonstrated that ondansetron intravenous injection of 4-8 mg reduced shivering during anesthesia without causing substantial hemodynamic abnormalities. The type of surgery, maximum sensory block following spinal anesthetic, period, and other factors all affect the frequency and intensity of shivering during and after the procedure [8]. After intrathecal injection, fentanyl has a quick onset and short duration in a cesarean major adverse effects [9,10].

The age demographics are as follows: 27.2% were 18-25 years, 58.8% were 26-36 years, and 14% were 36-41 years. Of the patients, 98.48% were overweight, and 1.51% had normal weight. The occurrence of overweight (BMI: 25-29.9 kg/m^2^) and obesity (BMI: 30 kg/m^2^) found in a 2015 national Iraq STEPS survey appears to be quite similar to previous local studies, such as in Erbil city [11].

Table 2 reveals that the frequency of incidences of shivering is considerably higher in the control group than in the fentanyl and ondansetron groups (p=0.0123). This result agrees with the study result of Jabalameli et al. [12], who found that in comparison to a placebo, ondansetron could considerably reduce postoperative shivering after craniotomy. Ondansetron has no effect on either core or peripheral temperature. PS was found in two (5%) patients in the ondansetron group, which was significantly lower than the control group's 10 (25%) patients (p=0.012). The incidence rate of postoperative shivering, despite being on ondansetron, was 0.41 (95%). The mean (standard deviation (SD)) central temperature in the ondansetron group was substantially higher in the preoperative time (36.6) than in the postoperative time (34.2) (p=0.001). Also, the preoperative mean peripheral temperature (36.5) was substantially greater than the postoperative (34.4) (p=0.001).

Shivering relief is faster and more effective with fentanyl than with butorphanol. During the commencement of shivering, mean arterial pressure, pulse rate, respiratory rate, and oxygen saturation all rise significantly, while core body temperature falls. Butorphanol produces more drowsiness, nausea, vomiting, and shivering than fentanyl. Fentanyl is more efficient and less time-consuming in preventing perioperative shivering than butorphanol [13]. Table 3 shows that 9 mg of bupivacaine resulted in a non-significant difference (p=0.987) in shivering between groups; however, the other doses resulted in a significant difference, with 10, 11, 12, and 13 mg doses exhibiting considerably higher shivering. The combination of 15 g of fentanyl and 1.8 mL of 0.5% bupivacaine (9 mg) was found to be an excellent dose combination for spinal anesthesia during cesarean section [14].

Introducing 20 μg fentanyl to 0.5% bupivacaine intrathecally dramatically decreased the frequency of shivering in lower limb orthopedic procedures under spinal anesthesia. The two drugs had no obvious differences [15]. Table 4 shows that the usage of 10 mg of ephedrine resulted in a non-significant difference (p=0.056) in shivering between groups; however, the other doses resulted in a significant difference, with the 15, 20, 25, and 30 mg doses exhibiting significantly increased shivering. This is in agreement with the study results of Ahmed [15], who divided participants into control group (group C), who received normal saline; group OL, who received 4 mg of intravenous ondansetron; and group OH, who received 8 mg ondansetron intravenously slowly [16]. The hemodynamic changes of the patients, motor block, the development of any problems, and the administration of pethidine and ephedrine were all assessed.

In the study by Mohamed et al. [6], in the ondansetron 4 mg group, approximately 53.3% and 46.7% received 10 and 12.5 mg, respectively. In the ondansetron 8 mg group, around 51.1% and 48.9% were given 12 and 15 mg, respectively. Shivering and vomiting were not observed in either group. The incidence of nausea differs between the studied groups in a statistically insignificant way. A prophylactic bolus of intravenous ondansetron (8 mg) and, to a lesser degree, 4 mg could reduce the decrease in mean blood pressure after spinal anesthesia, in addition to the required dose of ephedrine, potentially reducing neonatal acidosis related to ephedrine use [6].

The study has some limitations. The research was conducted at a single center, which may limit the generalizability of findings to other healthcare settings. The present study focused specifically on pregnant women undergoing cesarean sections, so the results may not be applied to other patient populations. The sample size of the study was relatively small, which could affect the statistical power of the study results. Only shivering during the surgical procedure was assessed, and postoperative shivering beyond the immediate intraoperative period was not considered. Therefore, additional research is needed to address these aspects comprehensively.

## Conclusions

When compared to the control group, the administration of two distinct intravenous doses of ondansetron and an intrathecal dose of fentanyl effectively reduces spinal-induced shivering. There is an increased incidence of shivering in patients who received higher doses of bupivacaine. Two different doses of ondansetron and fentanyl are administered, and when compared to the control group, they significantly lessen shivering brought on by spinal anesthesia.
